# Trends in lower extremity peripheral arterial disease incidence since 1990 and forecasting future statistics using Global Burden of Disease 2021: a time-series analysis

**DOI:** 10.3389/fpubh.2025.1521927

**Published:** 2025-04-09

**Authors:** Jiacheng Li, Chengxin Weng, Tiehao Wang, Wei Lu, Lihong Lin, Jiawen Wu, Guobing Cheng, Qiang Hu, Yi Guo

**Affiliations:** ^1^Department of Vascular Surgery, The Quzhou Affiliated Hospital of Wenzhou Medical University, Quzhou People’s Hospital, Quzhou, Zhejiang, China; ^2^Division of Vascular Surgery, Department of General Surgery, West China Hospital, Sichuan University, Chengdu, China; ^3^Department of Nosocomial Infection Control, The Quzhou Affiliated Hospital of Wenzhou Medical University, Quzhou People’s Hospital, Quzhou, Zhejiang, China

**Keywords:** autoregressive integrated moving average model, Global Burden of Disease, incidence, Joinpoint regression analysis, lower extremity peripheral arterial disease, time series

## Abstract

**Background:**

Lower extremity peripheral arterial disease (LEPAD) significantly affects quality of life and is associated with severe cardiovascular risks. Studies on its long-term incidence trends are limited.

**Objective:**

This study aims to analyze global trends in LEPAD incidence from 1990 to 2021 using the Global Burden of Disease (GBD) 2021 database and to forecast future trends between 2022 and 2030, providing insights for healthcare planning and resource allocation.

**Methods:**

Data were extracted from the GBD 2021 database by genders, age groups, continents, and sociodemographic index (SDI) levels. Using Joinpoint regression analysis, annual percentage changes (APC) and average annual percentage changes (AAPC) were calculated to assess age-standardized incidence rates (ASIR) historical trends. Autoregressive integrated moving average (ARIMA) model was applied to predict ASIR between 2022 and 2030.

**Results:**

The global ASIR of LEPAD showed a slight decrease from 1990 to 2021, though regional differences were notable. In 2021, the highest ASIR was in the Americas, while Africa had the lowest. Gender and age disparities were significant, and females and older populations were at higher risk. ARIMA predictions indicate a stable ASIR trend from 2025 onward.

**Conclusion:**

This study provides a comprehensive analysis of LEPAD incidence trends and a forecast through 2030. While global incidence may stabilize, the rising burden in lower-income countries calls for prioritizing early intervention and health education in high-risk regions. These findings emphasize the importance of targeted resource allocation and strategic prevention efforts.

## Background

Lower extremity peripheral arterial disease (LEPAD) is caused by plaque buildup in the peripheral vessels, of which the gold standard definition is having an ankle-brachial index (ABI) of less than 0.90, with leg pain on exertion called intermittent claudication in those with an ABI below that threshold (1), ranking as the 10th leading cause of mortality and the 11th leading cause of disability among cardiovascular diseases (2). The global prevalence of LEPAD has risen sharply, with an estimated 113.4 million people affected worldwide in 2019, and 10.5 million new cases annually (3). Despite some regions experiencing a declining age-standardized incidence rate (ASIR), the absolute number of cases continues to increase due to population aging and growing burden of metabolic risk factors, like diabetes, hypertension, and hyperlipidemia (4, 5). Disability-adjusted life years (DALYs) due to LEPAD also have increased significantly, with a 98% rise between 1990 and 2019 in the United States alone (2). Additionally, the economic burden of LEPAD is substantial, with estimated healthcare costs exceeding 21 billion USD annually, primarily due to hospitalizations, revascularization procedures, and long-term management of critical limb ischemia (6). Given its rising burden, LEPAD represents a critical challenge for public health systems worldwide, necessitating improved screening, early intervention, and targeted management strategies to reduce its long-term impact.

Global Burden of Disease (GBD) is a comprehensive global health assessment program initiative aimed at quantifying the impact of various diseases, injuries, and risk factors on global population health (7). This database encompasses data on disease burden from 1990 to 2021, including metrics such as incidence, prevalence, mortality, and DALYs, systematically providing insights into the global trends of various diseases and health risks. The GBD data are extensively applied in health policy development, resource allocation, and public health research. Among these metrics, incidence serves as a key measure of disease burden, reflecting the number of new cases per 100,000 population and helping to evaluate the current spread and trends of diseases, particularly in regions with significant shifts in population and age structures. Compared to prevalence and mortality, incidence offers unique value in assessing the effectiveness of health interventions, setting priorities for resource allocation, and formulating early prevention strategies. The ASIR, defined as the number of new cases per 100,000 people after age adjustment (8), removes the confounding effect of age structure, providing a clearer reflection of disease risk. ASIR is thus ideal for evaluating temporal trends of diseases at global or regional levels.

Currently, there is limited research on the long-term incidence trends of LEPAD and the application of predictive models (9). Accordingly, this study utilizes the latest data from the GBD 2021 to conduct a comprehensive analysis of LEPAD incidence over a 32-year period from 1990 to 2021. Metrics used include incidence and ASIR, describing the current distribution of LEPAD across different regions and populations, employing Joinpoint analysis to interpret the temporal trends of LEPAD’s ASIR from 1990 to 2021, and applying an autoregressive integrated moving average (ARIMA) model to predict changes in ASIR over the next 29 years. This study not only fills the knowledge gap regarding long-term LEPAD trends and regional disparities but also provides data-driven forecasts that can aid in prioritizing global health interventions and resource allocation in the future.

## Materials and methods

### Data source

The GBD 2021 database, led by the Institute for Health Metrics and Evaluation (IHME) at the University of Washington, is a comprehensive database that evaluates the global incidence, prevalence, years lived with disability (YLDs), DALYs, and healthy life expectancy (HALE) for 371 diseases and injuries across 204 countries and territories (10, 11). Given the heterogeneity in data availability across countries, particularly in low-resource settings, GBD employs multiple adjustment mechanisms to enhance reliability. These include DisMod-MR 2.1/3.0, a Bayesian meta-regression tool for integrating diverse data sources (12), hierarchical modeling and geospatial interpolation for imputing missing data (10), and cause-of-death ensemble modeling (CODEm) to correct for underreporting and misclassification (13). These rigorous adjustments improve data consistency and comparability across regions.

In this study, we extracted LEPAD ASIR data from GBD for 204 countries and territories in 2021, stratified by gender (male, female) and age groups (40–44 years, 45–49 years, 50–54 years, 55–59 years, 60–64 years, 65–69 years, 70+ years). Additionally, ASIR data from 1990 to 2021 were extracted at the global level, by gender (female, male), by continent (Africa, America, Asia, Europe), and by sociodemographic index (SDI) regions (low SDI, low-middle SDI, middle SDI, high-middle SDI, high SDI) with corresponding 95% uncertainty intervals (UI).

### Statistical analysis

#### Burden description

Using R Software (version 4.4.1) with packages including ggmap (14), sf (15), terra (16), maps (17), dplyr (18), and ggplot2 (19), we first described the distribution of LEPAD ASIR across regions in 2021. Subsequently, a bar chart was used to depict LEPAD incidence by age group within each gender group in 2021.

#### Joinpoint regression analysis

Joinpoint regression analysis was used to examine temporal trends, employing Joinpoint Regression Software (version 5.2.0), developed by the U.S. National Cancer Institute. The grid search method (GSM) was employed to detect the optimal number and locations of joinpoints, and the Monte Carlo permutation test was applied to statistically assess the significance of detected joinpoints, estimating their number, locations, and *p* values (20). The model selection was guided by the Bayesian Information Criterion (BIC), which balances model complexity and goodness-of-fit (21). To facilitate direct comparisons, we pre-specified the number of joinpoints as 3 in all regression analyses, ensuring that temporal trend variations were evaluated under standardized conditions. Log-linear models are particularly suitable for small sample sizes and can reduce estimation bias (22). Therefore, this study analyzed LEPAD incidence trends from 1990 to 2021 by establishing a log-linear model for ASIR. To verify the log-linearity assumption, we examined the relationship between ln (ASIR) and Year, of which the scatter plot (Supplementary Figure 1) exhibited a linear trend. The annual percentage change (APC) and average annual percentage change (AAPC) with corresponding 95% confidence intervals (CI) were calculated for each time trend of LEPAD’s ASIR. Subgroup analyses were conducted by gender, continent, and SDI to evaluate differences across groups.

#### ARIMA analysis

R Software (version 4.4.1) was used with packages including forestcast (23), tseries (24), and aTSA (25). We used ARIMA model to forecast ASIR between 2022 and 2030. The ARIMA model was constructed based on ASIR data reported from 1990 to 2021. The auto.arima() function (26) was employed to automatically select the autoregression order (AR (*p* = 0)), moving average order (MA (*q* = 2)), and the degree of difference (I (*d* = 2)) of the ARIMA model, which compares the combinatorial spectrum of parameters according to rule of the minimum Akaike’s Information Criterion (AIC) or BIC (27). Only the optimal model is available as an output, hence no other models were available to be recorded in the results. Residual analysis was performed using checkresiduals() function, which generated residuals plot, corresponding autocorrelation function (ACF) and histogram (Supplementary Figure 2). Additionally, Box-Ljung test confirmed that the residuals followed a white noise process ( $\chi^{2}=1.26$ , *p* = 0.999), indicting no significant autocorrelation.

Given that smoking is the most significant behavioral risk factor for LEPAD (28) and WHO’s Framework Convention on Tobacco Control (FCTC) (29) was introduced in 2003, a sensitivity analysis was performed by excluding data from 1990 to 2003.

To further validate the robustness of our findings, considering ARIMA’s limitations in capturing external risk factors, we conducted subgroup analyses by gender, continent, and SDI, which allowed us to assess whether forecasted ASIR trends exhibited variations across different demographic and economic contexts.

All tests were two-sided, with a significance level of *α* = 0.05.

## Results

### Burden of LEPAD in 2021

**Figure 1:**
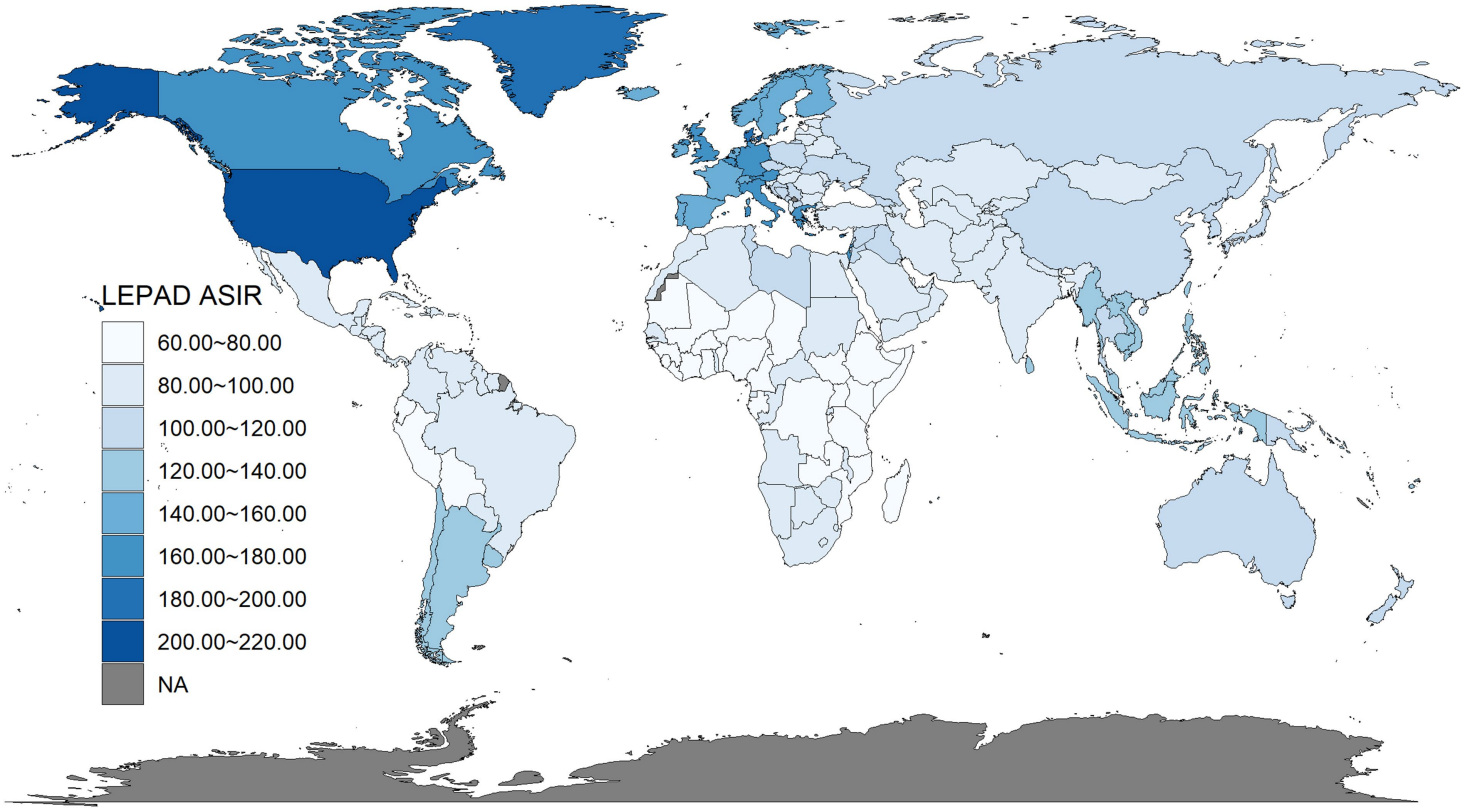
World map of ASIR of LEPAD in 2021.

In 2021, the global ASIR for LEPAD was 115.44 (95% UI: 100.04 to 132.72), with the highest ASIR observed in the America at 133.70 (95% UI: 115.15 to 154.43) and the lowest in Africa at 83.60 (95% UI: 71.72 to 97.00). ASIR varied considerably across countries (Figure 1), with the United States reporting the highest ASIR at 211.78 (95% UI: 189.72 to 235.81) and Ethiopia the lowest at 65.93 (95% UI: 56.53 to 76.75).

**Figure 2:**
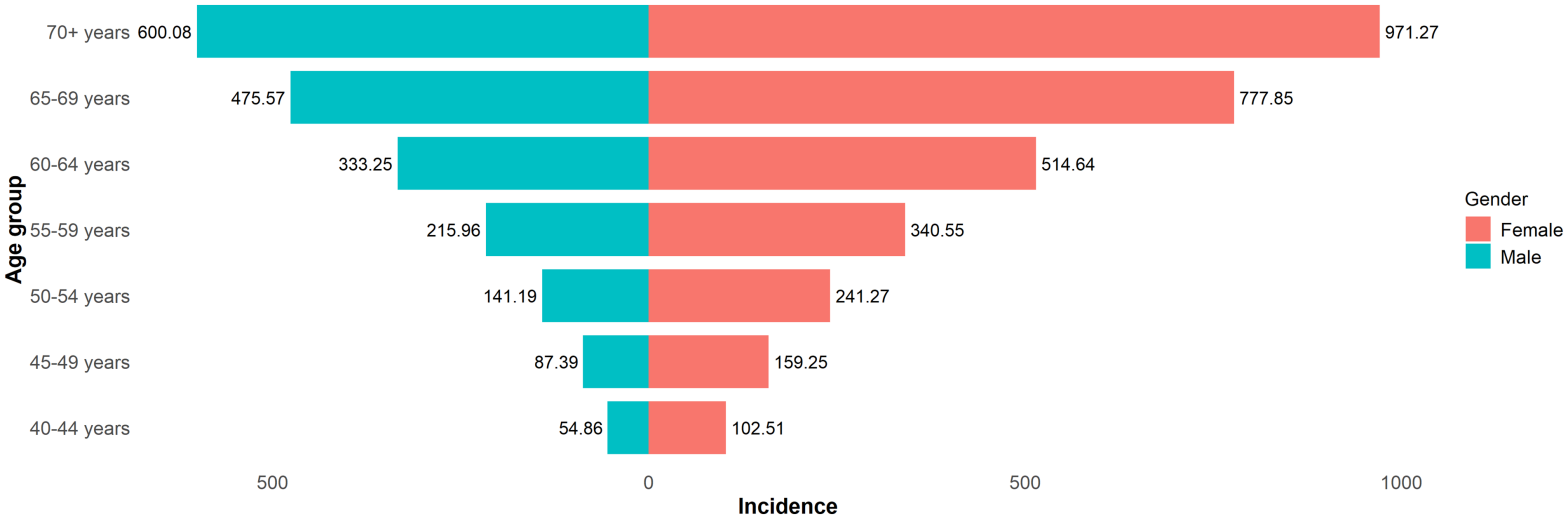
Age distribution of incidence of LEPAD of different genders.

Figure 2 illustrated the age distribution of LEPAD incidence by gender in 2021. Both gender groups showed an increasing trend in incidence with advancing age. For males, the lowest incidence was observed in the 40–44 age group (54.86, 95% UI: 44.09 to 67.69), while the highest was in those aged 70 and above (600.08, 95% UI: 475.97 to 735.53). Similarly, for females, the lowest incidence occurred in the 40–44 age group (102.51, 95% UI: 83.39 to 125.45), with the highest in those aged 70 and above (971.27, 95% UI: 776.80 to 1199.12). At each age level, the incidence for males was consistently lower than that for females.

### Joinpoint regression analysis for ASIR of LEPAD

**Figure 3:**
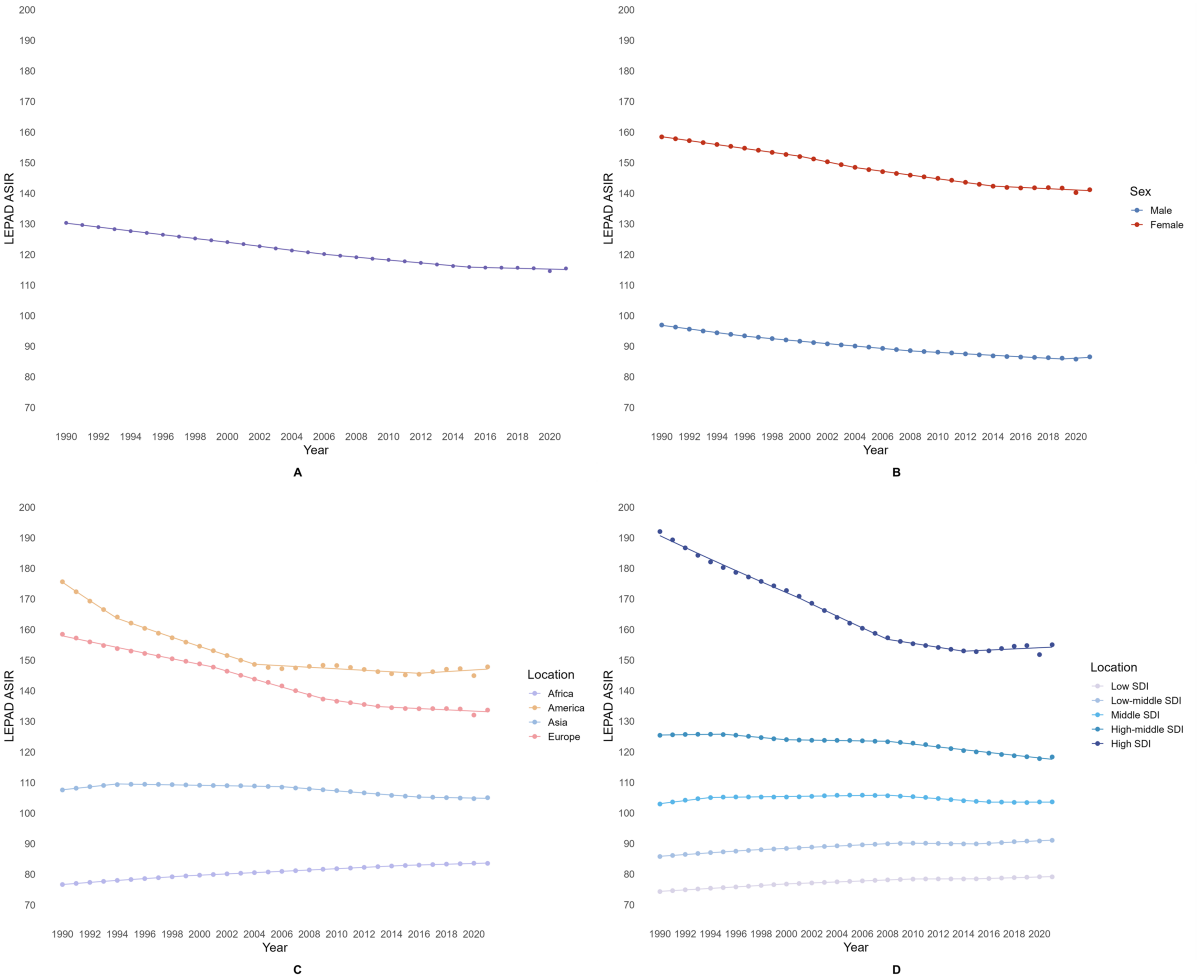
Trends in ASIR of LEPAD between 1990 and 2021 **(A)** Global; **(B)** Different Genders; **(C)** Different Continents; **(D)** Different SDI Regions.

The overall analysis showed a decreasing trend in LEPAD ASIR from 1990 to 2021, with an AAPC of −0.40 (95% CI: −0.43 to −0.37) (Figure 3A; Table 1). Subsequently, subgroup analyses were conducted by gender, continent, and SDI, with the results as follows.

**Table 1:** Joinpoint analysis for ASIR in 204 countries for years 1990–2021.

Classify	Trend 1			Trend 2			Trend 3			Trend 4			AAPC (95% CI)	*p*
	Years	APC (95% CI)	*p*	Years	APC (95% CI)	*p*	Years	APC (95% CI)	*p*	Years	APC (95% CI)	*p*		
Global	1990 ~ 2000	−0.49 (−0.52, −0.45)	<0.001	2000 ~ 2006	−0.54 (−0.63, −0.44)	<0.001	2006 ~ 2015	−0.40 (−0.44, −0.35)	<0.001	2015 ~ 2021	−0.11 (−0.19, −0.04)	0.005	−0.40 (−0.43, −0.37)	<0.001
Genders														
Male	1990 ~ 1996	−0.62 (−0.67, −0.57)	<0.001	1996 ~ 2008	−0.44 (−0.46, −0.42)	<0.001	2008 ~ 2019	−0.28 (−0.30, −0.25)	<0.001	2019 ~ 2021	0.31 (0.02, 0.60)	0.039	−0.37 (−0.39, −0.35)	<0.001
Female	1990 ~ 2000	−0.41 (−0.55, −0.31)	0.015	2000 ~ 2004	−0.61 (−0.76, −0.27)	<0.001	2004 ~ 2014	−0.42 (−0.50, −0.25)	0.013	2014 ~ 2021	−0.15 (−0.24, 0.08)	0.119	−0.38 (−0.39, −0.36)	<0.001
Continents														
Africa	1990 ~ 1993	0.45 (0.40, 0.50)	<0.001	1993 ~ 1999	0.38 (0.36, 0.40)	<0.001	1999 ~ 2015	0.26 (0.26, 0.27)	<0.001	2015 ~ 2021	0.15 (0.13, 0.17)	<0.001	0.28 (0.28, 0.29)	<0.001
America	1990 ~ 1994	0.44 (0.37, 0.51)	<0.001	1994 ~ 2006	−0.07 (−0.08, −0.05)	<0.001	2006 ~ 2016	−0.32 (−0.34, −0.30)	<0.001	2016 ~ 2021	−0.07 (−0.13, −0.02)	0.006	−0.08 (−0.10, −0.07)	<0.001
Asia	1990 ~ 1994	−1.73 (−2.28, −1.17)	<0.001	1994 ~ 2004	−0.95 (−1.11, −0.79)	<0.001	2004 ~ 2016	−0.17 (−0.26, −0.07)	0.001	2016 ~ 2021	0.19 (−0.12, 0.50)	0.223	−0.57 (−0.67, −0.47)	<0.001
Europe	1990 ~ 2001	−0.60 (−0.66, −0.55)	<0.001	2001 ~ 2009	−0.90 (−1.01, −0.79)	<0.001	2009 ~ 2013	−0.48 (−0.90, −0.05)	0.031	2013 ~ 2021	−0.16 (−0.25, −0.06)	0.002	−0.55 (−0.61, −0.48)	<0.001
SDI regions														
Low SDI	1990 ~ 2000	0.32 (0.31, 0.33)	<0.001	2000 ~ 2010	0.21 (0.20, 0.22)	<0.001	2010 ~ 2015	0.01 (−0.03, 0.05)	0.648	2015 ~ 2021	0.16 (0.13, 0.18)	<0.001	0.20 (0.19, 0.21)	<0.001
Low-middle SDI	1990 ~ 1997	0.33 (0.31, 0.35)	<0.001	1997 ~ 2009	0.22 (0.21, 0.23)	<0.001	2009 ~ 2015	−0.05 (−0.09, −0.01)	0.013	2015 ~ 2021	0.23 (0.20, 0.25)	<0.001	0.19 (0.18, 0.20)	<0.001
Middle SDI	1990 ~ 1994	0.49 (0.40, 0.58)	<0.001	1994 ~ 2008	0.05 (0.04, 0.07)	<0.001	2008 ~ 2016	−0.27 (−0.31, −0.23)	<0.001	2016 ~ 2021	0.00 (−0.06, 0.07)	0.951	0.02 (0.00, 0.03)	0.097
High-middle SDI	1990 ~ 1995	0.04 (−0.08, 0.17)	0.479	1995 ~ 2000	−0.29 (−0.47, −0.11)	0.003	2000 ~ 2008	−0.05 (−0.13, 0.03)	0.185	2008 ~ 2021	−0.38 (−0.41, −0.35)	<0.001	−0.21 (−0.25, −0.17)	<0.001
High SDI	1990 ~ 2001	−1.02 (−1.12, −0.93)	<0.001	2001 ~ 2008	−1.18 (−1.41, −0.94)	<0.001	2008 ~ 2014	−0.42 (−0.73, −0.10)	0.013	2014 ~ 2021	0.12 (−0.07, 0.32)	0.191	−0.68 (−0.77, −0.59)	<0.001

First, the gender subgroup analysis revealed that ASIR was consistently lower in males than in females across all years, with both groups showing an overall decline (Male’s AAPC: -0.37, 95% CI: −0.39 to −0.35; Female’s AAPC: -0.38, 95% CI: −0.39 to −0.36) (Figure 3B; Table 1).

Second, the continental subgroup analysis indicated a consistent ranking of ASIR among the four continents each year: the Americas had the highest ASIR, followed by Europe, Asia, and Africa with the lowest. Over the 32-year period, ASIR in Africa showed an overall increase (AAPC: 0.28, 95% CI: 0.28 to 0.29), while the other continents showed an overall decrease (Asia’s AAPC: -0.08, 95% CI: −0.10 to −0.07; America’s AAPC: -0.57, 95% CI: −0.67 to −0.47; Europe’s AAPC: -0.55, 95% CI: −0.61 to −0.48) (Figure 3C; Table 1).

Lastly, the SDI-based subgroup analysis demonstrated an increasing trend in ASIR with rising SDI levels each year. High-SDI regions consistently had the highest ASIR, followed by high-middle SDI, middle SDI, low-middle SDI, and low SDI regions with the lowest rates. The ASIR in middle SDI regions showed no significant change over the 32 years (AAPC: 0.02, 95% CI: 0.00 to 0.03), while ASIR in low SDI (AAPC: 0.20, 95% CI: 0.19 to 0.21) and low-middle SDI regions (AAPC: 0.19, 95% CI: 0.18 to 0.20) increased. In contrast, high-middle SDI (AAPC: -0.21, 95% CI: −0.25 to −0.17) and high SDI regions (AAPC: -0.68, 95% CI: −0.77 to −0.59) showed a decreasing ASIR trend (Figure 3D; Table 1).

### Forecast analysis for ASIR of LEPAD

**Figure 4:**
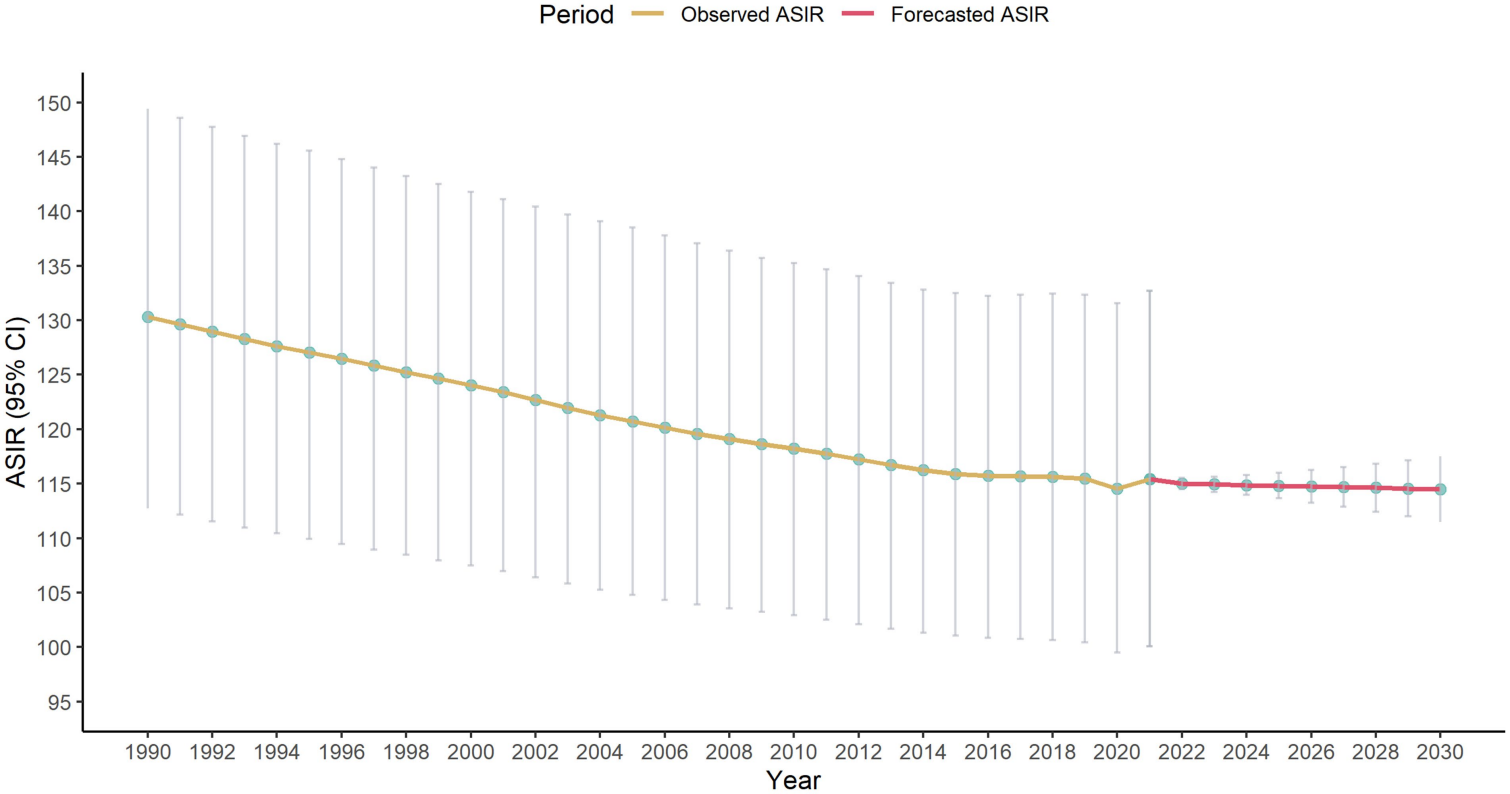
ASIR of LEPAD assessed from 1990 to 2021 alongside forecasted values for the period between 2022 and 2030.

Results of the ARIMA model were shown in Figure 4. The study forecasted that the ASIR of LEPAD would be 115.01 (95% CI: 114.48 to 115.54) in 2022, and would decrease to 114.50 (95% CI: 111.50 to 117.49) in 2030.

Sensitivity analysis of ARIMA model were shown in Supplementary Figure 3, which were not changed significantly compared to the main analysis. The study forecasted that the ASIR of LEPAD would be 115.00 (95% CI: 114.28 to 115.72) in 2022 and would decrease to 114.52 (95% CI: 110.76 to 118.28) in 2030.

**Figure 5:**
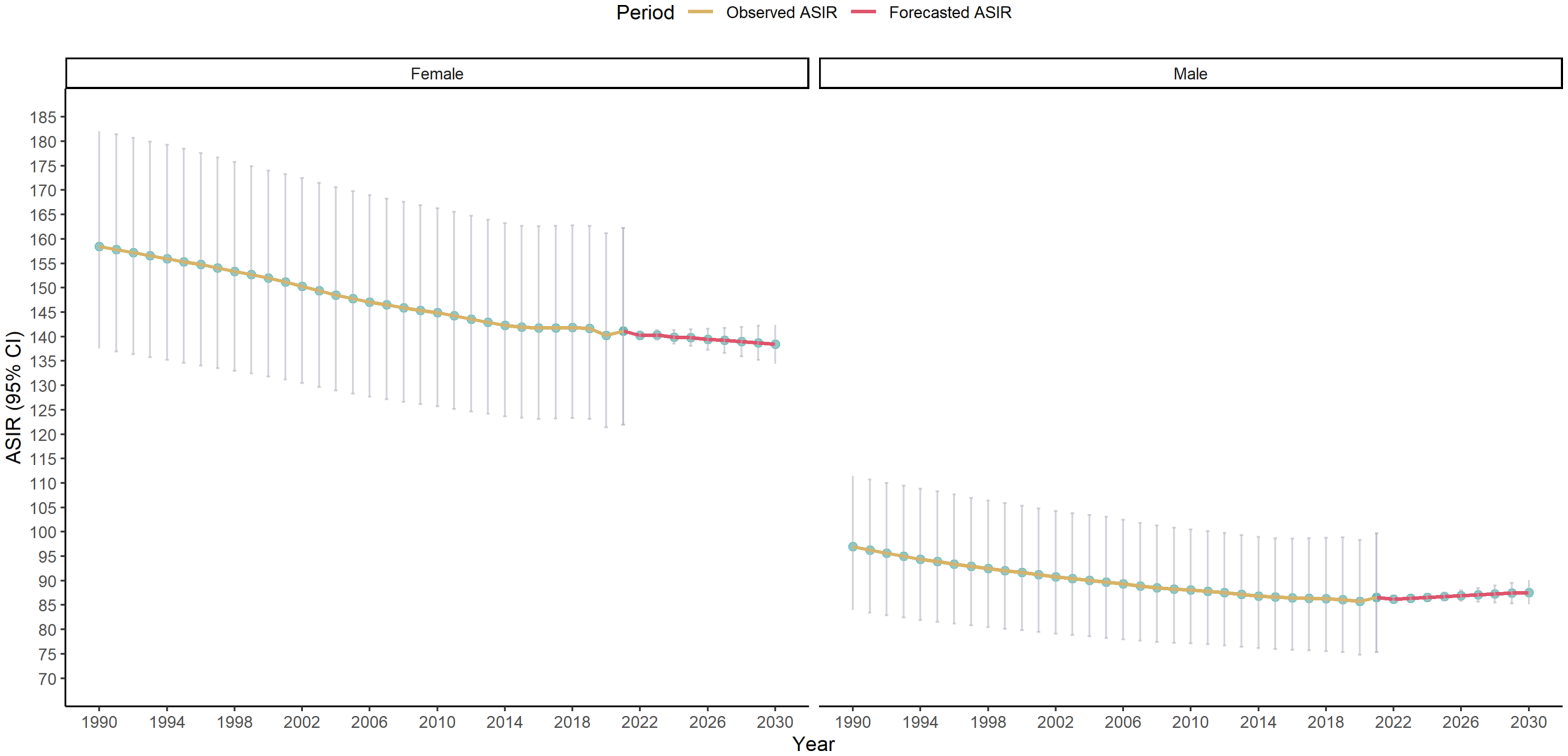
Gender subgroup analyses of ASIR of LEPAD Assessed from 1990 to 2021 alongside forecasted values for the period between 2022 and 2030.

**Figure 6:**
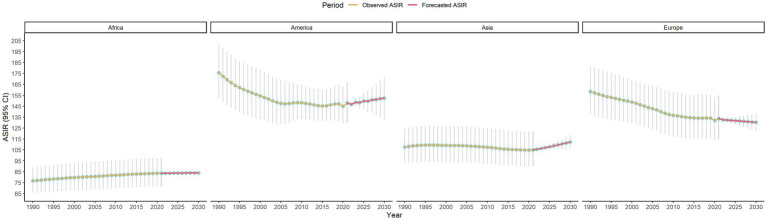
Continent subgroup analyses of ASIR of LEPAD assessed from 1990 to 2021 alongside forecasted values for the period between 2022 and 2030.

**Figure 7:**
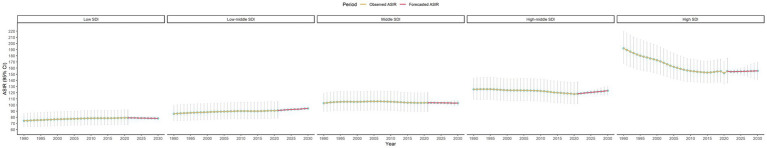
SDI subgroup analyses of ASIR of LEPAD assessed from 1990 to 2021 alongside forecasted values for the period between 2022 and 2030.

Subsequently, subgroup analyses were conducted by gender, continent, and SDI, with the results as follows. First, gender difference was shown in Figure 5. From 1990 to 2030 the ASIR for females remained consistently higher than for males. Both genders showed a declining trend, with a steeper decrease observed in males. The projected ASIR differences between genders persisted throughout the study period, indicating a stable yet distinct gender-based trend in LEPAD’s ASIR. Second, continent difference was shown in Figure 6. ASIR trends varied across continents. America has the highest ASIR, followed by Europe, Asia and Africa. All continents showed a general declining trend, with the steepest decrease in America and Europe. Third, SDI difference was shown in Figure 7. High SDI regions had the highest ASIR, while low SDI regions had the lowest. A general declining trend was observed across all groups, with the steepest reduction in high SDI regions. The ASIR differences among SDI groups persisted over time.

## Discussion

Although several studies have explored the epidemiological trends of LEPAD, limited research focused on the long-term incidence trends and the application of predictive models. For example, Fowkes et al. conducted a systematic review to analyze the burden of LEPAD across various socioeconomic and regional contexts, highlighting that existing data are insufficient for systematic future predictions, particularly regarding the disease burden in low-income and middle-income countries (30). Maheswaran et al. assessed the impact of national guidelines on LEPAD revascularization rate, focused on the treatment interventions and healthcare policies within National Healthcare System (NHS England) and indicated that the polices led to a reduction in revascularization rates, with a more pronounced impact in socioeconomically deprived areas (31). Compared with previous researches, this study conducted a comprehensive analysis of global LEPAD incidence trends from 1990 to 2021 using data from the GBD 2021 database and to predict future trends through the application of ARIMA models. This research has notable advantages in terms of data source and analytical approach, First, GBD 2021 dataset offered broad coverage and a long-time span. Second, this study employed an ARIMA model to forecast LEPAD ASIR, with the advantage of stabilizing time-series data and adjusting parameters to achieve precise future trend predictions, addressing gaps in predictive capabilities found in other studies. This study enhances the accuracy and broad applicability of LEPAD incidence trend analysis in terms of methodology and sample selection, thereby providing reliable support for the formulation of global public health strategies.

Our findings revealed substantial demographic differences in LEPAD incidence, with both gender and age playing crucial roles in disease burden and progression. LEPAD was more prevalent in females than in males, a trend that aligned with recent findings from the American Heart Association (AHA), which reported that women are more likely to develop LEPAD despite historically being underdiagnosed and undertreated (32). First, biological mechanisms largely account for this disparity. Estrogen plays an essential role in vascular homeostasis by enhancing nitric oxide bioavailability, reducing arterial stiffness, and suppressing inflammatory responses. Following menopause, estrogen deficiency leads to endothelial dysfunction, increased arterial calcification, and heightened oxidative stress, accelerating atherosclerosis progression (32). Smoking is a major modifiable risk factor that promotes oxidative stress, endothelial dysfunction, and inflammatory activation, leading to faster disease progression and worse post-procedural outcomes (28), which also amplifies this disparity, as women exhibit a stronger dose–response relationship between smoking and LEPAD than men (33). Additionally, women often exhibit atypical symptoms, such as leg fatigue, leading to underdiagnosis and treatment delays (31, 34). Disparities in healthcare access further compound these issues, with women being less likely to receive timely vascular assessments, optimal pharmacologic therapy, or revascularization procedures compared to men (35). Age is a key driver of LEPAD progression (36). Age-related arterial stiffness, endothelial dysfunction, and chronic low-grade inflammation further accelerate vascular deterioration (37). These findings underscore the importance of integrating demographic strategies into LEPAD management, including early screening for at-risk women, aggressive risk factor modification, and tailored rehabilitation programs to mitigate functional decline in older adult patients.

Despite the global decline in LEPAD incidence over the past 30 years, substantial regional disparities persist. Our study highlighted that while ASIR has decreased in high SDI regions, it has increased in Africa and other low SDI regions. This pattern aligns with a recent epidemiological study (9), which has shown a decline in LEPAD-related mortality in high-income countries since 2000, particularly among Black Americans and individuals in lower socioeconomic groups (38). Low SDI regions, particularly in Africa, are driven by multiple factors, including high smoking prevalence and inadequate tobacco control policies (39), which contributes to LEPAD progression. Strengthening tobacco control measures, such as higher excise taxes, public smoking bans, and stronger health education campaigns. Limited healthcare infrastructure further exacerbates the burden, leading to underdiagnosis and delayed intervention, as many patients lack access to primary care services and vascular specialists (40). To address this gap, ABI screening into primary care settings and developing mobile vascular screening units in underserved areas could facilitate earlier detection and timely management. Moreover, socioeconomic and racial disparities further amplify the LEPAD burden in resource-limited settings. Grant et al. showed that lower socioeconomic status is associated with worse LEPAD outcomes, with individuals in poverty-stricken areas experiencing higher rates of critical limb ischemia and increased mortality (41). Addressing these disparities requires expanding universal healthcare coverage, like cardiovascular medications and revascularization procedures, as well as community-based health education to improve disease awareness. These findings emphasize the urgent need for tailored regional strategies to reduce LEPAD burden. High SDI regions should continue investing in preventive strategies and early screening, while low SDI regions require enhanced healthcare accessibility and targeted risk factor modification programs.

The ARIMA model revealed gender and regional disparities in LEPAD incidence, underscoring the need for targeted intervention strategies. Women are projected to bear a greater LEPAD burden, reinforcing the necessity for earlier screening, targeted pharmacotherapy, and improved access to revascularization procedures (34). Additionally, low SDI regions continued to experience increasing ASIR, highlighting the urgent need for expanded screening, primary care and subsidized medication access (42). In high SDI regions, lipid control and structured rehabilitation should remain priorities. Given the projected future burden, LEPAD is expected to exert substantial pressure on healthcare resources (43). To effectively reduce the long-term impact, it is essential to prioritize early screening, public health education, and resource allocation in high-risk areas. Systematic screening and early prevention for high-risk populations, such as older individuals, smokers, and those with metabolic syndrome, can significantly reduce LEPAD incidence and disease progression (9). Lifestyle modifications, such as smoking cessation, increased physical activity, and blood glucose and pressure management, as preventive measures for high-risk individuals to lower their risk of developing LEPAD (44). Furthermore, establishing a management system for high-risk populations that provides long-term monitoring, follow-up, and health education may help reduce the incidence risk and alleviate the burden on healthcare systems (45).

This study still has several limitations. First, inherent limitations in the GBD database and the ARIMA forecasting approach restrict the ability to incorporate critical external variables, such as tobacco control policies. Since GBD data relies on modeled estimates rather than primary surveillance data, potential underreporting or misclassification biases may exist, particularly in low resource setting, like Somalia. Additionally, the ARIMA model relies on the stability and linear trend of time-series data, which may not fully capture the impact of sudden changes in public health policies and advancements in medical interventions (46). Although this study employed auto.arima() function to enhance data stability, limitations may still arise when analyzing long-term data (47, 48). Lastly, variations in data quality across regions within the GBD database may lead to biases in global incidence estimates (10, 49, 50).

## Conclusion

In conclusion, this study conducted an in-depth analysis of global LEPAD incidence trends using data from the GBD and employed an ARIMA model to forecast future trends, providing a scientific basis for public health management of LEPAD across different regions. The findings indicate that although the overall incidence of LEPAD is gradually decreasing, the burden continues to rise in low- and middle-income countries, necessitating prioritized attention and intervention. Based on these results, this study recommends prioritizing resource allocation in high-risk areas to strengthen screening, early intervention, and health education efforts to address the potential future burden of LEPAD. By integrating the latest mechanistic research and improvements in predictive models, future LEPAD management and prevention efforts will likely advance further, providing critical support for optimizing the global public health system.

The increasing LEPAD burden in low- and middle-income countries necessitates long-term cross-regional studies. Future efforts should focus on establishing global collaborative projects that improve data quality and regional trend validation. Moreover, future studies should focus on integrating real-world data from electronic health records, national registries, and large-scale cohort studies to validate and refine forecasting models. Enhancing the availability and accuracy of LEPAD epidemiological data in low resource areas will provide a stronger evidence base for tailored health policies and targeted prevention strategies. Additionally, refining forecasting methodologies is crucial for improving predictive accuracy, especially with constraints of data (48). Future research should explore hybrid forecasting approaches, including machine learning-enhanced time-series models and Bayesian frameworks.

## Data Availability

Publicly available datasets were analyzed in this study. This data can be found here: https://vizhub.healthdata.org/gbd-results/.
