# Supplementary material for: Trends in lower extremity peripheral arterial disease incidence since 1990 and forecasting future statistics using Global Burden of Disease 2021: a time-series analysis
Source: Front Public Health. 2025 Apr 9;13:1521927. doi: 10.3389/fpubh.2025.1521927 (PMC12014615; doi:10.3389/fpubh.2025.1521927)
Supplement: Supplementary file 1 [file Data_Sheet_1.docx]

**Supplementary Material**


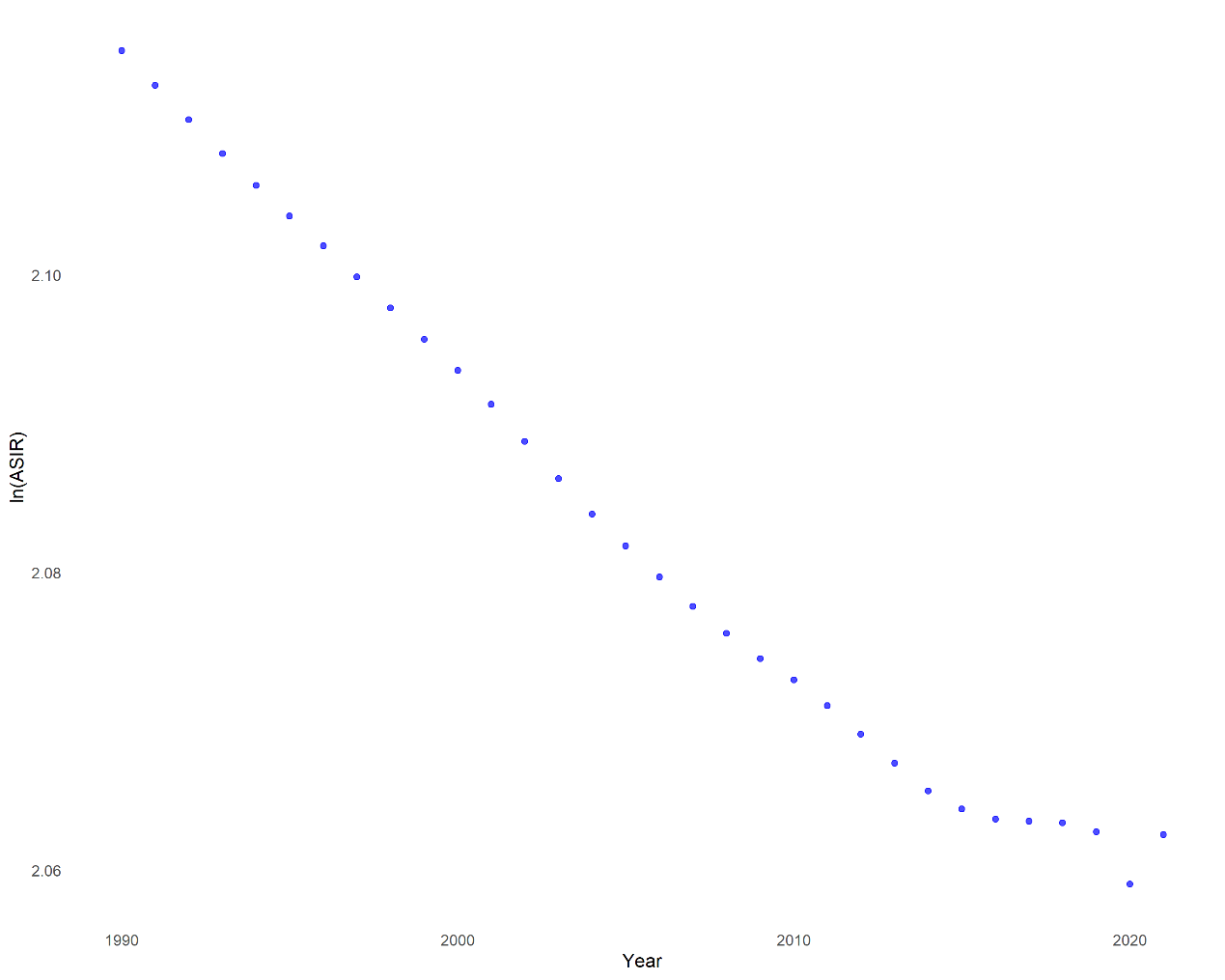


**Supplementary Figure 1. Scatter Plot of In(ASIR) v.s. Year**

**
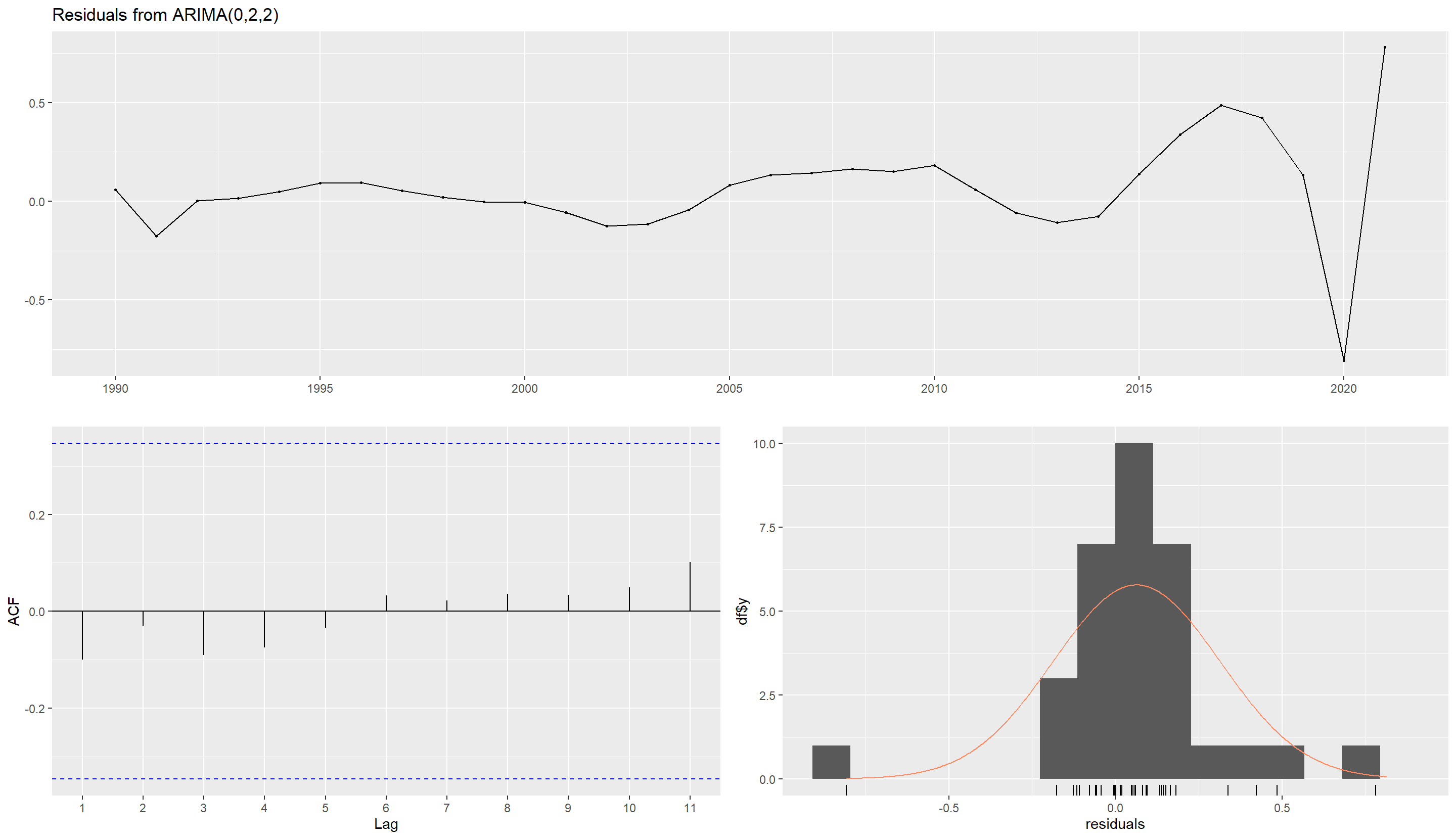
**

**Supplementary Figure 2. Residuals Test Results**

**
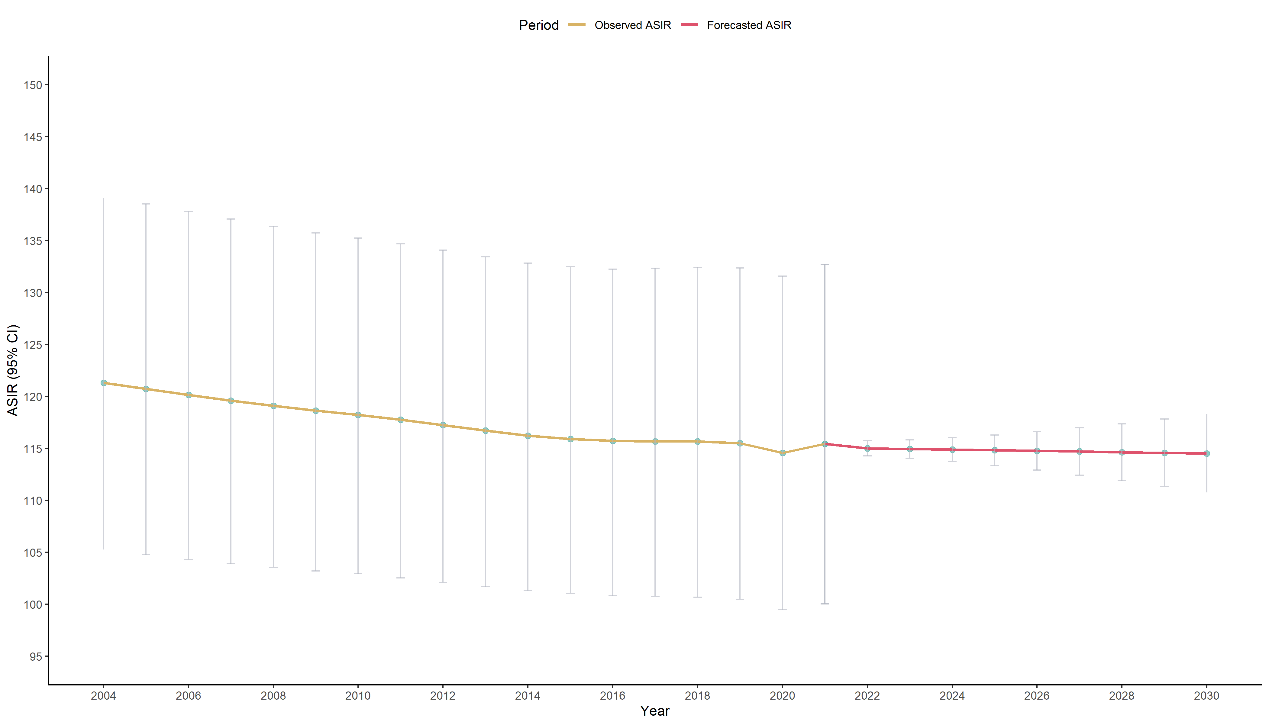
**

**Supplementary Figure 3. Sensitivity Analysis of ARIMA Model**
